# Self-control as an important factor affecting the online learning readiness of Vietnamese medical and health students during the COVID-19 pandemic: a network analysis

**DOI:** 10.3352/jeehp.2022.19.22

**Published:** 2022-08-25

**Authors:** Minh Tu Nguyen, Binh Thang Tran, Thanh Gia Nguyen, Minh Tri Phan, Thi Thu Tham Luong, Dinh Duong Le

**Affiliations:** 1 Office for Undergraduate Training, University of Medicine and Pharmacy, Hue University, Hue, Vietnam; 2 Faculty of Public Health, University of Medicine and Pharmacy, Hue University, Hue, Vietnam; 3 University of Medicine and Pharmacy, Hue University, Hue, Vietnam; Hallym University, Korea

**Keywords:** Communication, Distance education, Internet, Motivation, Self-control

## Abstract

**Purpose:**

The current study aimed to use network analysis to investigate medical and health students’ readiness for online learning during the coronavirus disease 2019 (COVID-19) pandemic at the University of Medicine and Pharmacy, Hue University.

**Methods:**

A questionnaire survey on the students’ readiness for online learning was performed using a Google Form from May 13 to June 22, 2021. In total, 1,377 completed responses were eligible for analysis out of 1,411 participants. The network structure was estimated for readiness scales with 6 factors: computer skills, internet skills, online communication, motivation, self-control, and self-learning. Data were fitted using a Gaussian graphical model with the extended Bayesian information criterion.

**Results:**

In 1,377 students, a network structure was identified with 6 nodes and no isolated nodes. The top 3 partial correlations were similar in networks for the overall sample and subgroups of gender and grade levels. The self-control node was the strongest for the connection to others, with the highest nodal strength. The change of nodal strength was greatest in online communication for both gender and grade levels. The correlation stability coefficient for nodal strength was achieved for all networks.

**Conclusion:**

These findings indicated that self-control was the most important factor in students’ readiness network structures for online learning. Therefore, self-control needs to be encouraged during online learning to improve the effectiveness of achieving online learning outcomes for students.

## Introduction

### Background/rationale

The current study was implemented during the fourth wave (from April 27, 2021 to June 22, 2022) of coronavirus disease 2019 (COVID-19) in Vietnam, which had the highest number of infected cases and most complicated situations compared to the previous waves [1]. COVID-19 caused a wide disruption of the traditional education model, and online learning became a crucial approach to adapt for all academic levels in Vietnam. Currently, a variety of evidence indicates that COVID-19 is deeply influencing all aspects of medical training education [2]. Similarly, the University of Medicine and Pharmacy, Hue University (Hue UMP) implemented different strategies to cope with the situation and maintain the education programs. Online learning has become a common method for both students and lecturers, who use a variety of digital platforms.

The literature shows that learners’ readiness is an essential issue in contributing to the success of online learning outcomes in higher education [3]. Students need to prepare to benefit from online learning settings [4]. Even though the online learning approach has existed for many years, it had not been implemented in many academic institutions before the COVID-19 pandemic, particularly in low-resource settings [5]. Moreover, learners’ readiness may impact the variability of resources, cultural backgrounds, disciplines, and prior academic qualifications. Therefore, students’ readiness needs to be evaluated to ensure the success, feasibility and suitability of online learning outcomes in this situation.

Network analysis has been applied to various psychological sciences such as depression, social anxiety disorder, and personality disorder [6]. In recent publications, the network structure was used to examine psychological factors related to academic pressure in medical students [7]. Similar to health psychology, the Online Learning Readiness Scale (OLRS) contains complex interactions between individual psychological factors (e.g., motivation, self-control, self-learning), environmental factors (e.g., communication), and individual capacities (e.g., computer skills or internet skills) [8]. A partial correlation (pc) network can predict the most important factor (nodes) and visualize the connections between nodes. Although many studies have been conducted on online learning during the COVID-19 pandemic, no study has been undertaken with a focus on network structure.

### Objectives

This study aimed to investigate the important factors in students’ readiness for online learning at a medical university in Vietnam using a network analysis of survey data.

## Methods

### Ethics statement

The proposed research and instruments were approved by the Ethics Committee for Biomedical Researches of the University of Medicine and Pharmacy, Hue University (approval no., H2021/421, dated August 12, 2021). Informed consent was obtained from the participants during the online survey.

### Study design

This study is a network analysis of data obtained through an online survey.

### Setting

Hue University of Medicine and Pharmacy provides 10 regular training undergraduate programs for medicine and health sciences, including medicine, pharmacy, odonto-stomatology, preventive medicine, nursing, midwifery, traditional medicine, public health, medical laboratory technology, and radiological technology. The annual enrollment number is about 7,000 per academic year. The survey questionnaire was distributed to a total of 2,000 students from May 13 to June 22, 2021.

### Participants

The initial student’ responses were 1,411 out of 2,000 target students, and we excluded 34 cases with incomplete information before data analysis. The students confirmed their participation on the first page before filling out the questionnaire. They received an electronic link via Google Forms by email, comprising a consent form and a study information sheet. The participating students were from 8 majors (Table 1).

### Variables

The variables for participants’ characteristics were age, sex, academic year, residence, monthly support, and their academic major. The variables of OLRS were computer skills, internet skills, online communication, self-learning, self-control, and online motivation.

### Data sources/measurement

Raw response data from participants are available from Dataset 1.

The structured questionnaire was created using a Google Form, including general characteristics and the University Students’ Readiness for E-Learning scale [8]. The measurement tool was used under the Creative Commons licenses CC-BY-ND. A survey questionnaire of 33 items is available in Supplement 1. The general characteristics were age, sex, academic year, residence, monthly support, and academic major. According to the current academic year of training, students were divided into 2 groups based on their grade levels: the lower grades were first and second-year students, while the higher grades included third-year or higher years.

The OLRS mentions a variety of aspects, including 6 domains with 33 items, comprising computer skills (5 items), internet skills (4 items), online communication (5 items), self-learning (8 items), self-control (4 items), and online motivation (7 items). A 5-point Likert scale is used to score each item, ranging from 1 (totally disagree) to 5 (totally agree). A higher score indicates better preparation for online learning during the COVID-19 pandemic.

### Bias

There was no bias in selecting target students. Only data from actual responses were used for the analysis.

### Study size

For network analysis, the study size could not be estimated because all responses were included.

### Statistical methods

The general characteristics of students were presented using numbers and percentages for categorical data and mean with standard deviation (SD) for continuous data. We tested the significance of differences between categorical variables (such as gender or grade levels) using the chi-square test or the Fisher exact test. All scores of total and 6 readiness domains had a skewed distribution according to the Shapiro-Wilk normality test. Then, we used nonparanormal (huge package ver. 1.3.5; https://www.rdocumentation.org/packages/huge/versions/1.3.5) transformation to normalize the readiness scales before estimating the networks. The network structure of the readiness scale was fitted using a Gaussian graphical model which wasregularized using the graphical lasso algorithm with the extended Bayesian information criterion (tuning=0.5) [9]. The network structure was estimated and plotted using the q-graph package (ver. 1.9; https://www.rdocumentation.org/packages/qgraph/versions/1.9.2). The network accuracy and stability were examined under bootstrapped 95% confidence intervals (CI) of edge-weights by the bootnet R package ver. 1.5 (The R Foundation for Statistical Computing, Vienna, Austria; https://www.r-project.org/). A correlation stability coefficient of at least 0.25 indicates the stability of the node centrality indices [9]. The network comparison test (NCT) was calculated to compare the differences between networks. The network characteristics were described by network density, global strength, averaged clustering coefficient, modularity index (Q), and average shortest path length. The statistical analyses were performed using the RStudio (2021. 9. 2; RStudio, Boston, MA, USA) and R ver. 4.1.2 (The R Foundation for Statistical Computing). The R code is available in Supplement 2.

## Results

### General characteristics and ORLS score

The overall characteristics were illustrated according to the total sample, gender and grade levels in Table 1. The mean age was 20.6 years, without a significant difference between male and female students. Most of the students were female (67.2%), first-year students (36.8%), and in the general doctor training program (47.5%). More than half of the students were from a city (54.4%) and had a middle range of monthly costs (US $ 111–156). In terms of OLRS, the total score differed significantly according to gender and grade level. Male and higher-grade students were more likely to have higher overall scores than female and lower-grade students. There was a statistically significant difference between male and female students in 3 dimensions of the OLRS: computer skills, internet skills, and online communication. Similarly, computer skills, online communication, and motivation were different between lower- and higher-grade levels.

### Network analysis

**Fig. 1.**
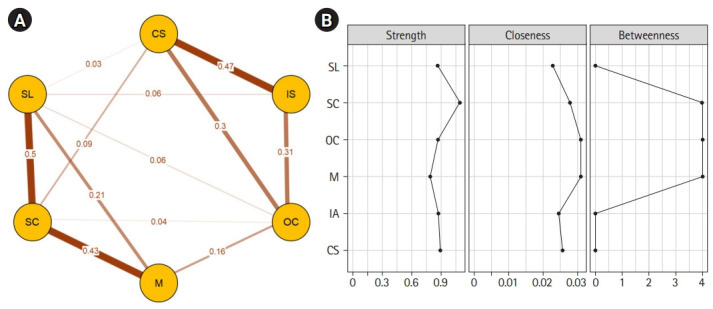
Network structure (A) and centrality indices (B) of the total sample. CS, computer skills; IS, internet skills; OC, online communication; M, online motivation; SC, self-control; SL, self-learning.

In the overall sample of 1,377 students, a network structure comprised 6 nodes without any isolated nodes. A total of 12 edges were created, and 5 edge weights had a pc of over 0.3 for the total sample network (Fig. 1). The strongest connection was observed for the relationship between self-learning and self-control (pc=0.50), followed by computer skills and internet skills (pc=0.48) and self-control and motivation (pc=0.43) (Supplement 3A).

**Fig. 2.**
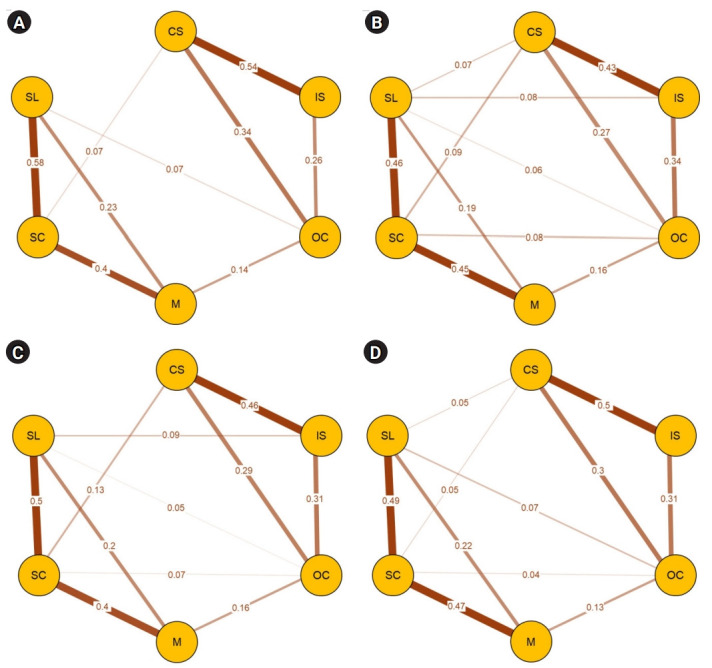
Network structures for male students (A), female students (B), lower-grade students (C), and higher-grade students (D). CS, computer skills; IS, internet skills; OC, online communication; M, online motivation; SC, self-control; SL, self-learning.

The network structures for gender and grade levels are drawn in Fig. 2. Similarly, the pc appeared to be strongest for the connection between self-control and self-learning in the 4 networks. In the female network, self-control was more strongly associated with motivation and was connected to online communication, whereas it was not in male students. The pc matrix of four network structures is presented in the supplemental tables (Supplement 3A–E).

**Fig. 3.**
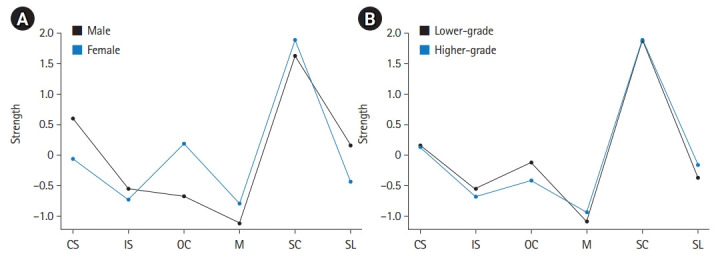
Nodal strength centrality indices for gender (A) and grade level (B). CS, computer skills; IS, internet skills; OC, online communication; M, online motivation; SC, self-control; SL, self-learning.

For the centrality indices, nodal strength, betweenness, and closeness were calculated and plotted in the supplemental materials (Supplement 4A–D). The highest nodal strength was observed for self-control in all networks (Fig. 3). The change in nodal strength was greatest for online communication for both gender and grade levels (Fig. 3, Supplement 3F).

The accuracy and central stability of networks were evaluated using the bootstrapped CIs of edge weights (Supplement 4E–N). Overall, the node strength was more stable than node closeness and betweenness for all networks (Supplement 4J–N). The correlation stability coefficient for nodal strength was achieved for all groups (Table 2).

## Discussion

### Key results

The current study analyzed a total of 1,377 students from 8 majors of medicine and health sciences to investigate the network structure of OLRS. We found that the overall score of OLRS had a statistically significant difference between male and female students. Regarding the sub-dimensions, computer skills, internet skills, and online communication were stronger in male students than in female students. The NCT results indicated a significant difference between male and female students (Supplement 3G). By contrast, self-control, self-learning, and study motivation were similar in terms of gender.

### Interpretation

In this study, we used network analysis as a new perspective on exploring how network structures differ and which dimensions are more strongly connected or more important than others [10]. A literature review has shown that the OLRS has multiple dimensions and varies among studies [11]. These findings generally suggest an equal global network structure for the total population, gender, and grade levels. We found a good network structure, with 6 nodes, and it was maintained for all networks. We found that the partial pairwise correlation was highest for the connection between self-control and self-learning, followed by that between computer skills and internet skills and the connection between self-control and study motivation.

Regarding the local network characteristics, centrality measures are the most common way to measure local network characteristics.

Our findings indicated that the self-control node was the strongest for the connection to others, with the highest nodal strength for all the networks. Some previous studies reported that self-control is an important component of online learning readiness among students. Horzum et al. [12] suggested that learner control explained 58% of the variance of online learning readiness by itself. This could be understood as indicating that learners’ self-control contributes directly to the effects of learning in a new environment. The COVID-19 pandemic has encouraged the utilization of these technologies and the distance learning approach for academic education. Besides the obvious benefits of online learning, learners will be influenced by numerous challenges in the online environment. Moreover, learner control is an important indicator of online learning success [4]. Therefore, students’ self-control is a critical factor in their online learning process.

The readiness scale was evaluated for different aspects of students, and it focused on the individual self-efficacy for online learning. The current study may contain several limitations that need to be considered and mentioned. Firstly, even though the OLRS instrument was tested and thoroughly reviewed for use, the validated process in the new situation may have affected the results. Secondly, the behavior and attitude of students in enrollment also influenced their answers, and unbalanced responses, particularly regarding the grade levels of participants, may have contributed to noise in these findings. Thirdly, we only included the OLRS scores of students, without relevant factors that may directly influence online learning, such as the teaching or the infrastructure of the online learning process. Although lecture-based teaching is easily transitioned to an online format, interactive small group sessions and clinical exposure are not as easily replicated in the COVID-19 pandemic. Despite these gaps, our study is one of the first works to investigate the OLRS using the network structure in a difficult situation.

### Comparison with previous studies

These findings differed slightly from the study of Hung et al. [11], which indicated that gender did not show significant differences for any of the OLRS dimensions, but they are consistent with the findings of higher scores for male students in all aspects. A recently published work confirmed that female students responded more positively to OLRS than male students [13]. The results of Rafique et al. [14] showed that a significant difference in opinions appeared on computer/internet self-efficacy and online communication self-efficacy based on respondents’ gender. Several pieces of evidence suggest that female students are more likely to have higher achievement learning outcomes because they tend to be more persistent, committed and have stronger self-regulation than males [15].

Grade level is directly related to competencies and influences online learning readiness outcomes. The current results are consistent with the previous studies in that the higher-grade students were more likely to have a higher score in all dimensions [11]. Moreover, we found significant differences between grade levels in computer skills, online communication, and the study motivation of participants. Rafique et al. [14] suggested that students’ grade strongly influenced their OLRS and led to significant differences in computer, internet, and online communication self-efficacy and learning motivation.

Hung et al. [11] suggested that self-directed learning and learner control influence study plans based on students’ knowledge and expectation for learning, the creation of learning outcomes given their pace and needs, participation, and responsibility in the decision-making process for student learning. The difference in nodal strength was greatest for online communication for both gender and grade levels. The current evidence suggests that communication contributes as an essential factor for learner readiness in online learning. The change in learning methods requires interaction between students and lecturers through digital devices [8]. Therefore, Kusel et al. [4] concluded that students’ communication capacity is a critical competency and an influential predictor of learning results.

### Limitations

Although the sampling was not randomized, the participation of 19.7% of all students in the University of Medicine and Pharmacy, Hue University may provide enough power to represent the status of students in this university.

### Generalizability

The results of this study may be generalizable to medical and health students in Vietnam.

### Conclusion

The present study illustrates that the top 3 pairwise correlations were self-control and self-learning, self-control and study motivation, and computer skills and internet skills for all network structures. These findings indicate that self-control was the most important factor in the network structures of students' readiness for online learning. Online communication had the largest difference in node strength between subgroups of gender and grade levels. We highly recommend encouraging self-control during online learning to increase the effectiveness of achieving online learning outcomes for students.

## Figures and Tables

**Table 1.** Demographic characteristics and students’ readiness scale according to gender and grade level

Characteristic	Total (n=1,377)	Male (n=451)	Female (n=926)	P-value	Lower grade (n=652)	Higher grade (n=725)	P-value
Age (yr)	20.6±1.73	20.7±1.87	20.5±1.66	0.116	19.1±0.98	21.8±1.21	<0.001
Gender							0.360
Male	451 (32.8)				222 (34.0)	229 (31.6)	
Female	926 (67.2)				430 (66.0)	496 (68.4)	
Academic years							
First	507 (36.8)	160 (35.5)	347 (37.5)	0.007			
Second	145 (10.5)	62 (13.7)	83 (8.96)				
Three	371 (26.9)	101 (22.4)	270 (29.2)				
Fourth	184 (13.4)	68 (15.1)	116 (12.5)				
Fifth	170 (12.3)	60 (13.3)	110 (11.9)				
Majors				NA			<0.001
Medicine	654 (47.5)	295 (65.4)	359 (38.8)		347 (53.2)	307 (42.3)	
Odonto-stomatology	127 (9.2)	43 (9.53)	84 (9.07)		54 (8.28)	73 (10.1)	
Traditional medicine	105(7.6)	28 (6.21)	77 (8.32)		38 (5.83)	67 (9.24)	
Preventive medicine	83 (6.0)	18 (3.99)	65 (7.02)		18 (2.76)	65 (8.97)	
Pharmacy	146 (10.6)	29 (6.43)	117 (12.6)		41 (6.29)	105 (14.5)	
Medical technicians^a)^	102 (7.4)	21 (4.66)	81 (8.75)		55 (8.44)	47 (6.48)	
Nursing	147 (10.7)	14 (3.1)	133 (14.4)		94 (14.4)	53 (7.31)	
Public health	13 (0.9)	3 (0.67)	10 (1.08)		5 (0.77)	8 (1.10)	
Residence				0.002			0.550
City	749 (54.4)	221 (49.1%)	523 (56.7)		345 (53.1)	399 (55.3)	
Rural	501 (36.4)	194 (43.1%)	307 (33.3)		247 (38.0)	254 (35.2)	
Mountainous	127 (9.2)	35 (7.78%)	92 (9.98)		58 (8.92)	69 (9.56)	
Monthly support (US $)				<0.001			0.648
<67	202 (14.7)	58 (12.9)	144 (15.6)		103 (15.8)	99 (13.7)	
67–<111	324 (23.5)	77 (17.1)	247 (26.7)		156 (23.9)	168 (23.2)	
111–<156	567 (41.2)	198 (43.8)	369 (39.8)		263 (40.3)	304 (41.9)	
>156	284 (20.6)	118 (26.2)	166 (17.9)		130 (19.9)	154 (21.2)	
Readiness scale							
Computer skills	18.6±4.30	19.7±4.34	18.1±4.19	<0.001	18.1±4.45	19.1±4.11	<0.001
internet skills	17.0±3.04	17.5±3.05	16.8±3.01	<0.001	16.8±3.17	17.1±2.91	0.141
Communication	18.7±4.28	19.2±4.29	18.5±4.26	0.004	18.4±4.34	19.1±4.20	<0.001
Motivation	23.4±5.79	23.4±6.25	23.4±5.56	0.879	22.9±5.74	23.9±5.80	<0.001
Self-control	13.3±3.10	13.4±3.20	13.2±3.05	0.216	13.3±3.08	13.3±3.12	0.965
Self-learning	28.3±6.04	28.5±6.45	28.2±5.83	0.198	28.3±6.19	28.3±5.90	0.958
Total score	119.0±21.1	122±21.5	118±20.9	0.004	118±21.7	121±20.6	0.007

Values are presented as mean±standard deviation or number (%). NA, not available.

a) Including the laboratory and image diagnostics program.

**Table 2.** Correlation stability coefficient of centrality indices according to gender and grade level

Correlation stability coefficient	Male	Female	Lower-grade	Higher-grade
Strength	0.361	0.438	0.439	0.439
Closeness	0.206	0.205	0.051	0.128
Betweenness	0.129	0.127	0	0.128

## Footnotes

**Data availability**
Data files are available from Harvard Dataverse: https://doi.org/10.7910/DVN/VWA2CT Dataset 1. Response data file from the participants, including general characteristics and the University Students’ Readiness for E-Learning scale.

## Supplementary materials

Supplementary files are available from Harvard Dataverse: https://doi.org/10.7910/DVN/VWA2CT

Supplement 1. Survey questionnaire used for the measurement of the Online Learning Readiness Scale.

Supplement 2. R code for the network analysis.

Supplement 3. Supplementary tables. (A) Correlation matrix of the network in the total sample. (B) Correlation matrix of the network in male students. (C) Correlation matrix of the network in female students. (D) Correlation matrix of the network in lower-grade students. (E) Correlation matrix of the network in higher-grade students. (F) Comparison of nodal strength according to gender and grade level. (G) Global connectivity of the estimated network structures.

Supplement 4. Supplementary figures. (A) Centrality indices of the network for male students. (B) Centrality indices of the network for female students. (C) Central indices of the network for lower-grade students. (D) Central indices of the network for higher-grade students. (E) Bootstrapped confidence intervals (CIs) of estimated edge weights for the total sample. (F) Bootstrapped CIs of estimated edge-weights for male students. (G) Bootstrapped CIs of estimated edge-weights for female students. (H) Bootstrapped CIs of estimated edge-weights for lower grade students. (I) Bootstrapped CIs of estimated edge-weights for higher grade students. (J) Case-dropped bootstraps’ centrality indices for the total sample. (K) Case-dropped bootstraps’ centrality indices for male students. (L) Case-dropped bootstraps’ centrality indices for female students. (M) Case-dropped bootstraps’ centrality indices for lower-grade students. (N) Case-dropped bootstraps’ centrality indices for higher-grade students.

Supplement 5. Audio recording of the abstract.
