# Supplementary material for: Self-control as an important factor affecting the online learning readiness of Vietnamese medical and health students during the COVID-19 pandemic: a network analysis
Source: J Educ Eval Health Prof. 2022 Aug 25;19:22. doi: 10.3352/jeehp.2022.19.22 (PMC9582298; doi:10.3352/jeehp.2022.19.22)
Supplement: Supplementary file 2 — Supplement 1. Survey questionnaire used for the measurement of the Online Learning Readiness Scale. [file jeehp-19-22-suppl1.docx]

**Supplement 1.** Survey questionnaire was used for the measurement of the Online Learning Readiness Scale

**Online Learning Readiness Scale**

**The questions are rated on a 5-point Likert scale:**

1. Totally disagree/never

2. Disagree/rarely

3. No opinion/normal

4. Agree/regularly

5. Totally agree/always

|  | | **1** | | **2** | **3** | **4** | **5** |
| --- | --- | --- | --- | --- | --- | --- | --- |
| **Computer skills** | |  | |  |  |  |  |
| **Q1** | I can easily use Windows operating systems. | 1 | | 2 | 3 | 4 | 5 |
| **Q2** | I can view the contents of an electronic file (sound, music, text, etc.) on the computer. | 1 | | 2 | 3 | 4 | 5 |
| **Q3** | I can solve the problems I encountered when using a computer. | 1 | | 2 | 3 | 4 | 5 |
| **Q4** | I can easily use the Office programs (Word, Excel, PowerPoint, Outlook, etc.). | 1 | | 2 | 3 | 4 | 5 |
| **Q5** | I can easily use the application softwares I need (editor, design, etc.). | 1 | | 2 | 3 | 4 | 5 |
| **Internet skills** | |  | |  |  |  |  |
| **Q6** | I can easily use Web browsers (Internet Explorer, Google Chrome, etc.). | 1 | | 2 | 3 | 4 | 5 |
| **Q7** | I can easily use search engines (Google, Yandex, etc.). | 1 | | 2 | 3 | 4 | 5 |
| **Q8** | I can download a file on the internet to my computer. | 1 | | 2 | 3 | 4 | 5 |
| **Q9** | I can easily access the information I seek on the internet. | 1 | | 2 | 3 | 4 | 5 |
| **Online communication** | |  | |  |  |  |  |
| **Q10** | I can use Internet tools (e-mail, discussion forums, Skype, etc.) to communicate effectively with people. | 1 | | 2 | 3 | 4 | 5 |
| **Q11** | I can easily ask questions in the internet forum discussions. | 1 | | 2 | 3 | 4 | 5 |
| **Q12** | I can express myself easily in written communication (emotions, humor, etc.). | 1 | | 2 | 3 | 4 | 5 |
| **Q13** | I can ask for help using internet tools (forum discussion sites, social networks, e-mail, etc.) to seek answers. | 1 | | 2 | 3 | 4 | 5 |
| **Q14** | I can communicate easily with voice or video on the internet (Skype, Google Hangout, Google Talk, etc.). | 1 | | 2 | 3 | 4 | 5 |
| **Self learning** | |  |  | |  |  |  |
| **Q15** | I determine my learning needs | 1 | | 2 | 3 | 4 | 5 |
| **Q16** | I set my learning objectives myself. | 1 | | 2 | 3 | 4 | 5 |
| **Q17** | I make my study plan myself when learning. | 1 | | 2 | 3 | 4 | 5 |
| **Q18** | I am fully committed to my own study plan when learning. | 1 | | 2 | 3 | 4 | 5 |
| **Q19** | I organize my current study plan according to new conditions. | 1 | | 2 | 3 | 4 | 5 |
| **Q20** | I identify appropriate sources and tools in the learning process. | 1 | | 2 | 3 | 4 | 5 |
| **Q21** | I believe that I am responsible for my own learning. | 1 | | 2 | 3 | 4 | 5 |
| **Q22** | I keep my learning desire high until learning is realized. | 1 | | 2 | 3 | 4 | 5 |
| **Self control** | |  | |  |  |  |  |
| **Q23** | I direct my learning process when learning an online subject. | 1 | | 2 | 3 | 4 | 5 |
| **Q24** | I decide how intensely I will concentrate on the learning materials on the internet. | 1 | | 2 | 3 | 4 | 5 |
| **Q25** | I decide when to study online learning materials. | 1 | | 2 | 3 | 4 | 5 |
| **Q26** | I decide for myself which order to work with online learning materials. | 1 | | 2 | 3 | 4 | 5 |
| **Online motivation** | |  | |  |  |  |  |
| **Q27** | I am eager to learn lessons on the internet. | 1 | | 2 | 3 | 4 | 5 |
| **Q28** | I am interested in learning lessons on the internet. | 1 | | 2 | 3 | 4 | 5 |
| **Q29** | Learning the lessons on the internet is an effective way to learn. | 1 | | 2 | 3 | 4 | 5 |
| **Q30** | I think it is be fun learning lessons on the internet. | 1 | | 2 | 3 | 4 | 5 |
| **Q31** | I am self-confident in learning lessons on the internet. | 1 | | 2 | 3 | 4 | 5 |
| **Q32** | I like to share my opinions with others when learning on the internet. | 1 | | 2 | 3 | 4 | 5 |
| **Q33** | I learn from my mistakes learning on the internet. | 1 | | 2 | 3 | 4 | 5 |
