# Supplementary material for: Self-control as an important factor affecting the online learning readiness of Vietnamese medical and health students during the COVID-19 pandemic: a network analysis
Source: J Educ Eval Health Prof. 2022 Aug 25;19:22. doi: 10.3352/jeehp.2022.19.22 (PMC9582298; doi:10.3352/jeehp.2022.19.22)
Supplement: Supplementary file 4 — Supplement 3. Supplementary tables. (A) Correlation matrix of the network in the total sample. (B) Correlation matrix of the network in male students. (C) Correlation matrix of the network in female students. (D) Correlation matrix of the network in lower-grade students. (E) Correlation matrix of the network in higher-grade students. (F) Comparison of nodal strength according to gender and grade level. (G) Global connectivity of the estimated network structures. [file jeehp-19-22-suppl3.docx]

**Supplement 3.** Supplementary tables

(A) Correlation matrix of the network in the total sample

|  | CS | IS | OC | M | SC | SL |
| --- | --- | --- | --- | --- | --- | --- |
| CS |  | 0.476 | 0.302 | 0.000 | 0.091 | 0.029 |
| IS | 0.476 |  | 0.000 | 0.313 | -0.023 | 0.069 |
| OC | 0.302 | 0.313 |  | 0.156 | 0.044 | 0.059 |
| M | 0.000 | 0.000 | 0.156 |  | 0.434 | 0.208 |
| SC | 0.091 | -0.023 | 0.044 | 0.434 |  | 0.501 |
| SL | 0.029 | 0.068 | 0.059 | 0.208 | 0.501 |  |

CS, computer skills; IS, internet skills; OC, online communication; M, online motivation; SC, self-control; SL, self-learning.

(B) Correlation matrix of the network in male students

|  | CS | IS | OC | M | SC | SL |
| --- | --- | --- | --- | --- | --- | --- |
| CS |  | 0.535 | 0.340 | 0.000 | 0.064 | 0.000 |
| IS | 0.535 |  | 0.263 | 0.000 | 0.000 | 0.028 |
| OC | 0.340 | 0.263 |  | 0.144 | 0.000 | 0.068 |
| M | 0.000 | 0.000 | 0.144 |  | 0.402 | 0.228 |
| SC | 0.064 | 0.000 | 0.000 | 0.402 |  | 0.573 |
| SL | 0.000 | 0.028 | 0.068 | 0.228 | 0.573 |  |

CS, computer skills; IS, internet skills; OC, online communication; M, online motivation; SC, self-control; SL, self-learning.

(C) Correlation matrix of the network in female students

|  | CS | IS | OC | M | SC | SL |
| --- | --- | --- | --- | --- | --- | --- |
| CS |  | 0.424 | 0.272 | 0.030 | 0.077 | 0.077 |
| IS | 0.424 |  | 0.335 | 0.000 | 0.000 | 0.062 |
| OC | 0.271 | 0.335 |  | 0.154 | 0.075 | 0.064 |
| M | 0.030 | 0.000 | 0.154 |  | 0.441 | 0.190 |
| SC | 0.077 | 0.000 | 0.075 | 0.441 |  | 0.453 |
| SL | 0.077 | 0.062 | 0.064 | 0.190 | 0.453 |  |

CS, computer skills; IS, internet skills; OC, online communication; M, online motivation; SC, self-control; SL, self-learning.

(D) Correlation matrix of the network in lower grade students

|  | CS | IS | OC | M | SC | SL |
| --- | --- | --- | --- | --- | --- | --- |
| CS |  | 0.447 | 0.288 | 0.020 | 0.118 | 0.024 |
| IS | 0.447 |  | 0.310 | 0.000 | 0.000 | 0.074 |
| OC | 0.288 | 0.310 |  | 0.166 | 0.057 | 0.054 |
| M | 0.020 | 0.000 | 0.166 |  | 0.392 | 0.204 |
| SC | 0.118 | 0.000 | 0.057 | 0.392 |  | 0.494 |
| SL | 0.024 | 0.074 | 0.054 | 0.204 | 0.494 |  |

CS, computer skills; IS, internet skills; OC, online communication; M, online motivation; SC, self-control; SL, self-learning.

(E) Correlation matrix of the network in higher grade students

| Nodes | CS | IS | OC | M | SC | SL |
| --- | --- | --- | --- | --- | --- | --- |
| CS |  | 0.495 | 0.300 | 0.000 | 0.044 | 0.054 |
| IS | 0.49 |  | 0.307 | 0.000 | 0.000 | 0.028 |
| OC | 0.301 | 0.307 |  | 0.122 | 0.042 | 0.079 |
| M | 0.000 | 0.000 | 0.122 |  | 0.463 | 0.224 |
| SC | 0.045 | 0.000 | 0.042 | 0.463 |  | 0.486 |
| SL | 0.054 | 0.028 | 0.079 | 0.224 | 0.486 |  |

CS, computer skills; IS, internet skills; OC, online communication; M, online motivation; SC, self-control; SL, self-learning.

(F) Comparison of nodal strength by gender and grade levels

| Nodes | Total | Gender | | | Grade levels | | |
| --- | --- | --- | --- | --- | --- | --- | --- |
|  |  | Male | Female | Change | Lower grade | Higher grade | Change |
| CS | -0.038 | 0.588 | -0.057 | -0.645 | 0.164 | 0.156 | -0.008 |
| IS | -0.221 | -0.565 | -0.745 | -0.181 | -0.537 | -0.648 | -0.111 |
| OC | -0.272 | -0.689 | 0.173 | 0.862 | -0.086 | -0.383 | -0.297 |
| M | -1.034 | -1.107 | -0.813 | 0.294 | -1.055 | -0.896 | 0.159 |
| SC | 1.919 | 1.616 | 1.885 | 0.270 | 1.857 | 1.895 | 0.038 |
| SL | -0.354 | 0.157 | -0.444 | -0.601 | -0.342 | -0.123 | 0.219 |

CS, computer skills; IS, internet skills; OC, online communication; M, online motivation; SC, self-control; SL, self-learning.

(G) Global connectivity of the estimated network structures

| Testing | Sexes | | | Groups | | |
| --- | --- | --- | --- | --- | --- | --- |
|  | Male (n=451) | Female (n=926) | P-value^a)^ | Lower-grade (n=652) | Higher-grade (n=725) | P-value |
| Density | 0.667 | 0.867 | 0.033 | 0.867 | 0.80 | 0.516 |
| Global strength | 2.645 | 2.65 | 0.867 | 2.65 | 2.646 | 0.938 |
| Average clustering coefficient | 0.50 | 0.90 | 0.014 | 0.900 | 0.844 | 0.760 |
| Modularity quality index (Q) | 0.385 | 0.297 | 0.024 | 0.306 | 0.360 | 0.094 |
| Average shortest path length | 1.333 | 1.133 | 0.025 | 1.133 | 1.200 | 0.773 |

^a)^Calculated by the permutation test (global strength from network comparison test and others from NetworkToolbox).
