# Supplementary material for: Self-control as an important factor affecting the online learning readiness of Vietnamese medical and health students during the COVID-19 pandemic: a network analysis
Source: J Educ Eval Health Prof. 2022 Aug 25;19:22. doi: 10.3352/jeehp.2022.19.22 (PMC9582298; doi:10.3352/jeehp.2022.19.22)
Supplement: Supplementary file 5 — Supplement 4. Supplementary figures. (A) Centrality indices of the network for male students. (B) Centrality indices of the network for female students. (C) Central indices of the network for lower-grade students. (D) Central indices of the network for higher-grade students. (E) Bootstrapped confidence intervals (CIs) of estimated edge weights for the total sample. (F) Bootstrapped CIs of estimated edge-weights for male students. (G) Bootstrapped CIs of estimated edge-weights for female students. (H) Bootstrapped CIs of estimated edge-weights for lower grade students. (I) Bootstrapped CIs of estimated edge-weights for higher grade students. (J) Case-dropped bootstraps’ centrality indices for the total sample. (K) Case-dropped bootstraps’ centrality indices for male students. (L) Case-dropped bootstraps’ centrality indices for female students. (M) Case-dropped bootstraps’ centrality indices for lower-grade students. (N) Case-dropped bootstraps’ centrality indices for higher-grade students. [file jeehp-19-22-suppl4.docx]

**Supplement 4.** Supplementary figures


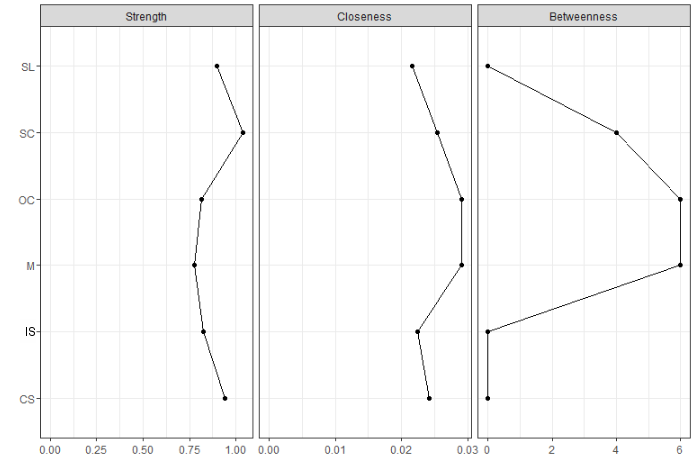


(A) Centrality indices of the network for male students. CS, computer skills; IS, internet skills; OC, online communication; M, online motivation; SC, self-control; SL, self-learning.


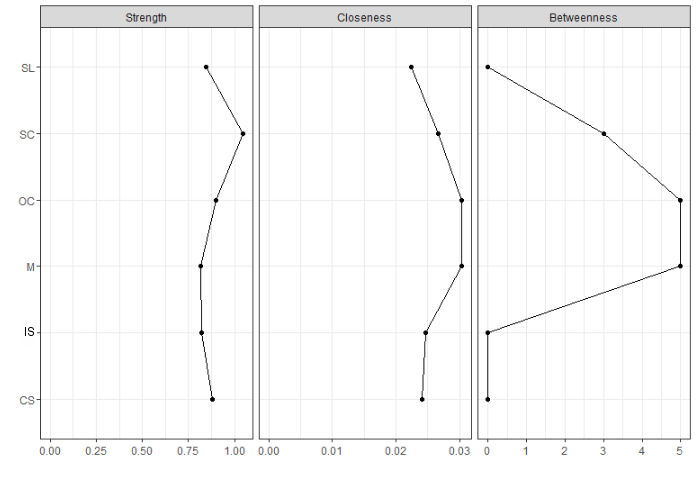


(B) Centrality indices of the network for female students.


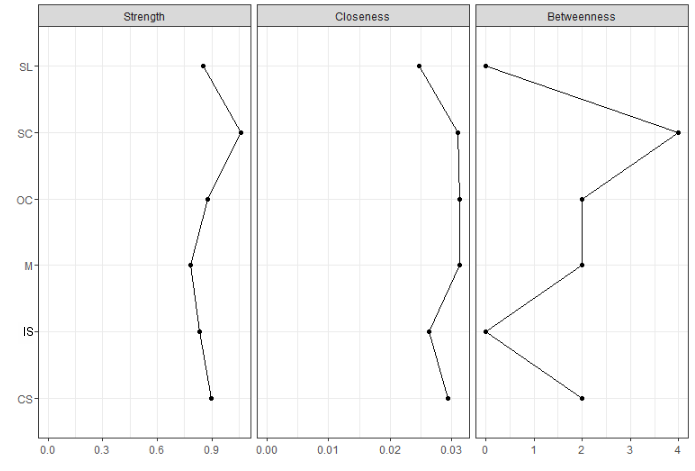


(C) Central indices of the network for lower grade students.


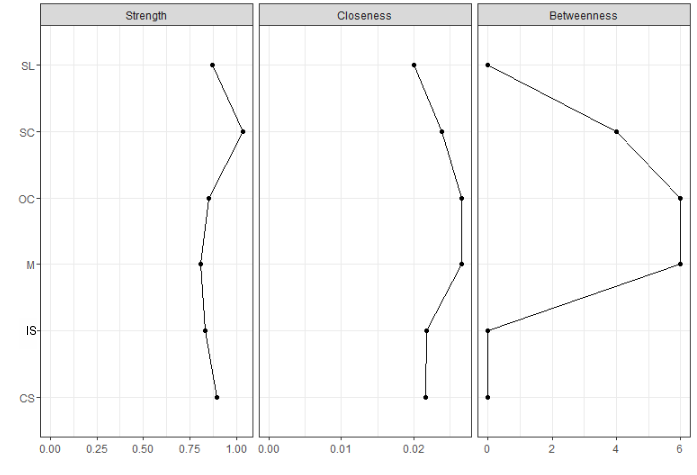


(D) Central indices of the network for higher grade students.


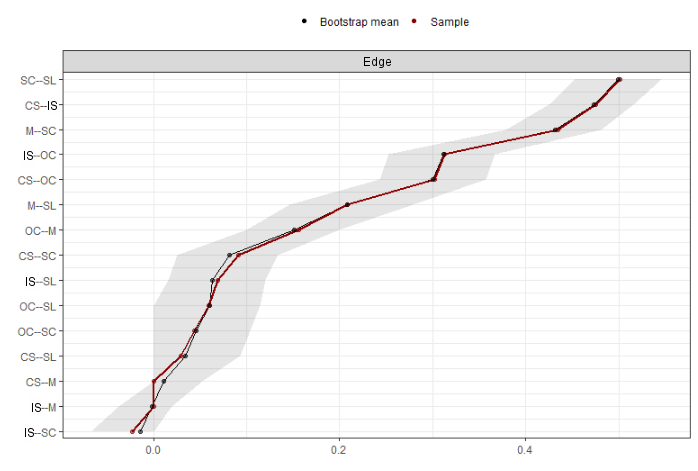


(E) Bootstrapped confidence intervals (CIs) of estimated edge weights for the total sample.


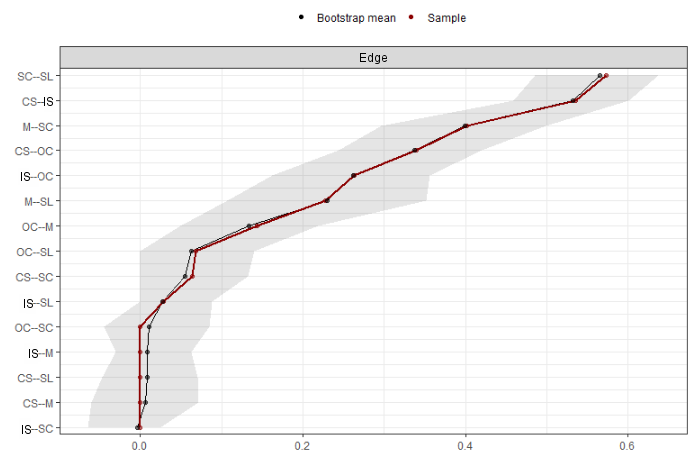


(F) Bootstrapped CIs of estimated edge-weights for male students.


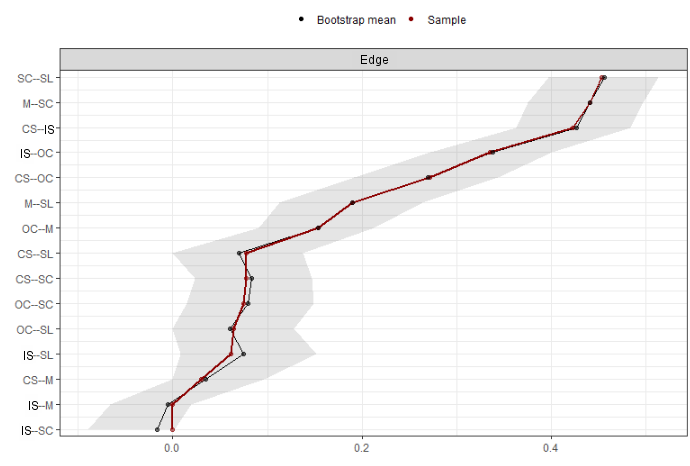


(G) Bootstrapped CIs of estimated edge-weights for female students.


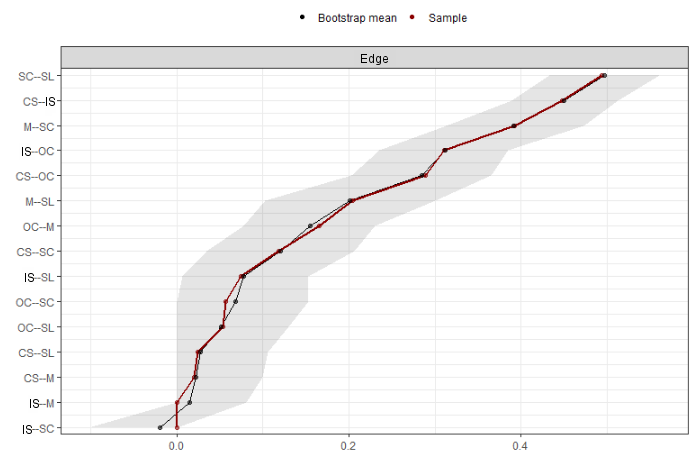


(H) Bootstrapped CIs of estimated edge-weights for lower grade students.


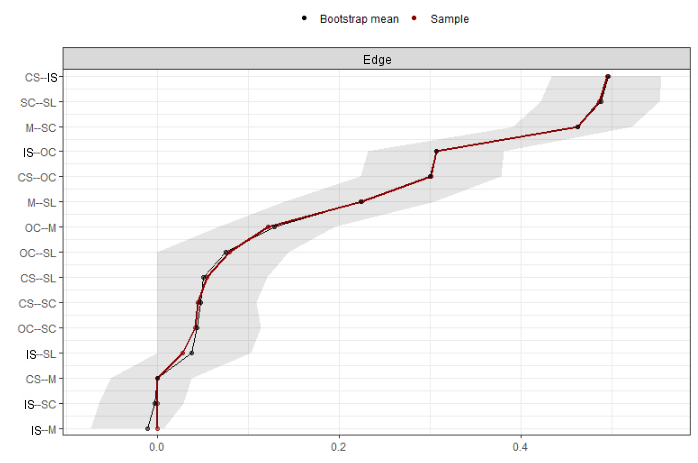


(I) Bootstrapped CIs of estimated edge-weights for higher grade students.


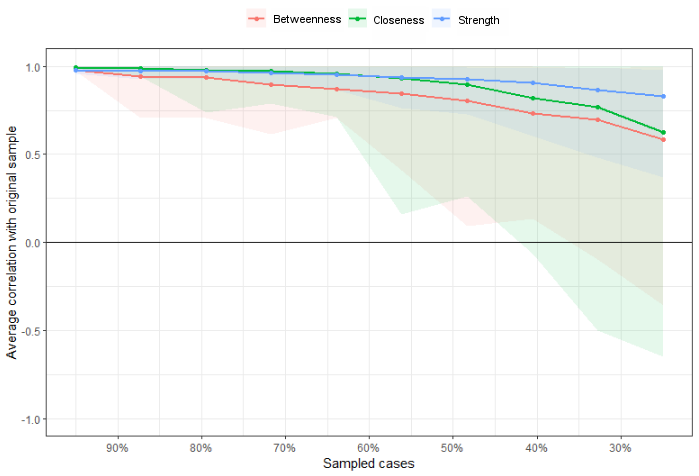


(J) Case-dropped bootstraps’ centrality indices for total sample.


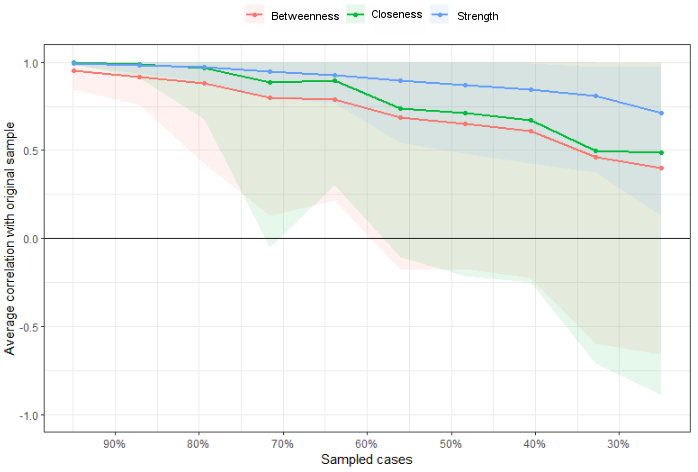


(K) Case-dropped bootstraps’ centrality indices for male students.


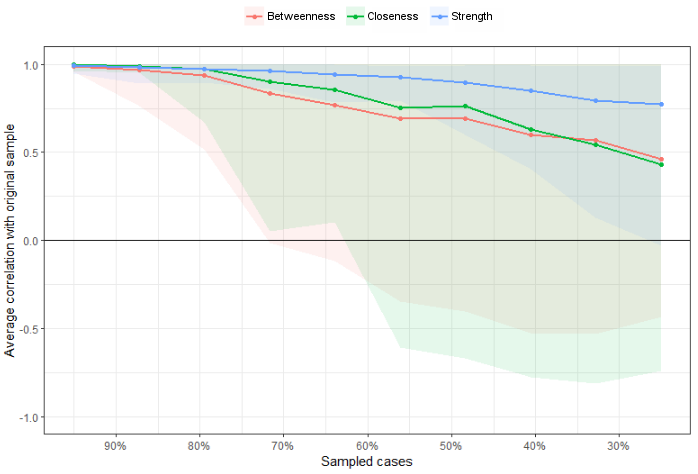


(L) Case-dropped bootstraps’ centrality indices for female students.


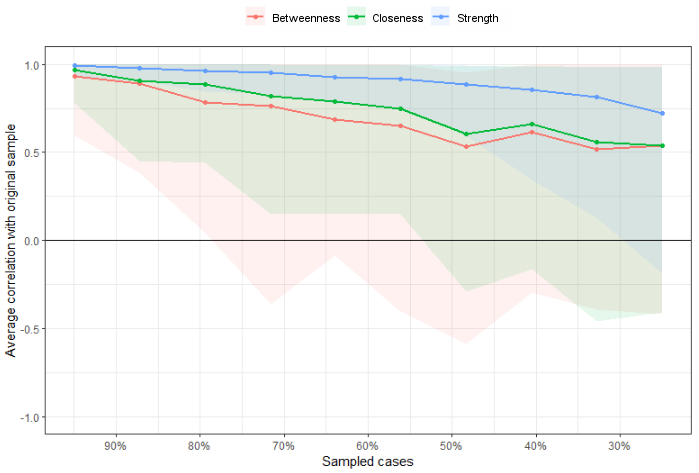


(M) Case-dropped bootstraps’ centrality indices for lower grade students.


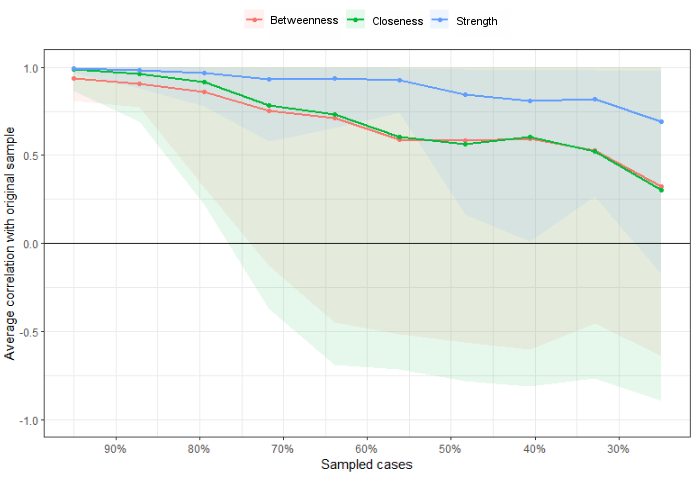


(N) Case-dropped bootstraps’ centrality indices for higher grade students.
